# Characterization of the complete chloroplast genome of *Coelogyne fimbriata* (Orchidaceae)

**DOI:** 10.1080/23802359.2020.1827058

**Published:** 2020-10-07

**Authors:** Qiu-Ping Wu, Jun-Wen Zhai, Kun-Lin Wu, Lin Fang, Song-Jun Zeng, Lin Li

**Affiliations:** a Key Laboratory of Plant Resources Conservation and Sustainable Utilization, South China Botanical Garden, Chinese Academy of Sciences, Guangzhou, China; b University of Chinese Academy of Sciences, Beijing, China; c Key Laboratory of National Forestry and Grassland Administration for Orchid Conservation and Utilization at College of Landscape Architecture, Fujian Agriculture and Forestry University, Fuzhou, China

**Keywords:** *Coelogyne fimbriata*, complete chloroplast genome, Illumina sequencing, Orchidaceae, phylogeny

## Abstract

*Coelogyne fimbriata* has been classified as a national second-class protected orchid species in China. In this study, we report and characterize the complete chloroplast (cp) genome sequence of *C*. *fimbriata* in an effort to provide genomic resources useful for promoting its conservation and systematic research. The complete genome is 159,010 bp in length and the overall GC content is 43.3%. The cp genome sequence has a typical quadripartite structure, comprising two inverted repeats (IRA and IRB) regions, which are separated by a small single**-**copy (SSC) region and a large single**-**copy (LSC) region. Moreover, a total of 135 functional genes were annotated, including 89 protein-coding genes, 38 tRNA genes, and 8 rRNA genes. The phylogenetic analysis recovered a close relationship between *C. fimbriata* and *Pleione formosana*, and both species are placed within the tribe Arethuseae (Orchidaceae).

*Coelogyne fimbriata* Lindl. (Orchidaceae) is native to a wide range of countries from Nepal, Bhutan, southern Fujian, Guangdong, Guangxi, Guizhou, Hainan, Hong Kong, Jiangxi, Sichuan, southeastern Xizang, Yunnan of China, northeastern India, Myanmar, Thailand, Vietnam, Cambodia, Laos to Peninsular Malaysia. This orchid grows in more humid areas on trees and rock walls, which has ovoid to ellipsoid pseudobulbs that carry two apical, oblong-elliptic or lanceolate leaves, and blooms in the fall arising on the newest mature pseudobulb. It has yellowish-green flowers with reddish-brown markings. The epithet refers to the fimbriate margins of the lip of the flower (Clayton 2002).

*Coelogyne fimbriata* has been classified as a national second-class protected orchid species in the Information System of Chinese Rare and Endangered Plants (ISCRPE; http://www.iplant.cn/rep/prot/Coelogyne). It is urgent to design effective conservation strategies for Chinese *C. fimbriata* populations because southern China is the northernmost edge of its distribution region where the populations are subject to ecological marginality and vulnerable to disturbance (Channell and Lomolino 2000; Swarts and Dixon 2009). Furthermore, given that *C. fimbriata* is so variable in morphology that scientists have defined many as different species (Gravendeel et al. 2001), it is more necessary to protect the germplasm resources of this species.

The samples of *C. fimbriata* were collected from Nanling National Nature Reserve, Northwest Guangdong, China (112.35E, 24.40N) and cultivated in the greenhouse of South China Botanical Garden, Guangzhou, China (113.36E, 23.18N). The voucher specimen (WuQiup018) was deposited in the herbarium of South China Botanical Garden (IBSC). Total genome DNA was extracted with the Trelief plant genomic DNA kit (TsingKe Biological Technology, Beijing, China) and tested for gel electrophoresis and concentration detection.

The high-throughput sequencing of the plastid genomes was performed using the Illumina Hiseq 2000 at the Beijing Genomics Institute (Beijing, China). A total of 1.73GB clean data were used for assembling the sequence as described by Jin et al. (2018). The plastid genome sequences of *Pleione bulbocodioides* (KY849819.1), *Pleione formosana* (MK361027.1), *Bletilla ochracea* (KT695602.1), and *Bletilla striata* (KT588924.1) were used as references to annotate the plastome using the PGA (Qu et al. 2019). Geneious R11 (Kearse et al. 2012) was used to check the accuracy of the assembly, and to adjust the start/stop codons and intron/exon boundaries of the annotation. The raw sequencing reads used in this study has been deposited in SRA with the accession number SRR12560166 and the annotated chloroplast (cp) genome sequence has been deposited into the GenBank with the accession number MT705722.

The complete cp genome sequence of *C*. *fimbriata* was found to be 159,010 bp in length. A total of 135 genes were annotated, including 89 protein-coding genes, 38 tRNA genes, and 8 rRNA genes. It has a typical quadripartite structure containing two short inverted repeat (IRA and IRB) regions (26,350 bp, each), separated by a small single-copy (SSC) region (18,835 bp) and a large single-copy (LSC) region (87,475 bp). The overall GC content is 43.3%, and the A, T, C, G base composition of the genome is 30.9, 31.7, 19, and 18.4%, respectively.

To investigate the phylogenetic position of *C. fimbriata*, a maximum-likelihood (ML) phylogenetic tree was constructed in IQ-TREE 1.6.12 with default parameters (Nguyen et al. 2015), based on 19 complete plastid genome sequences downloaded from the GenBank (Figure 1). We conducted sequences alignment using the MAFFT v.7.0.17 (Katoh and Standley 2013) plugin in Geneious, using *Goodyera fumata* (GenBank: NC_026773) and *Anoectochilus emeiensis* (GenBank: NC_033895) as outgroups. Our phylogenetic analyses indicate that *C. fimbriata* and *P. formosana* form a well-supported sister lineage, with *Bletilla formosana* as a successive closer taxon within the tribe Arethuseae. The complete cp genome of *C. fimbriata* will contribute to the development of conservation strategy for this rare orchid, as well as for further phylogenetic studies.

**Figure 1.**
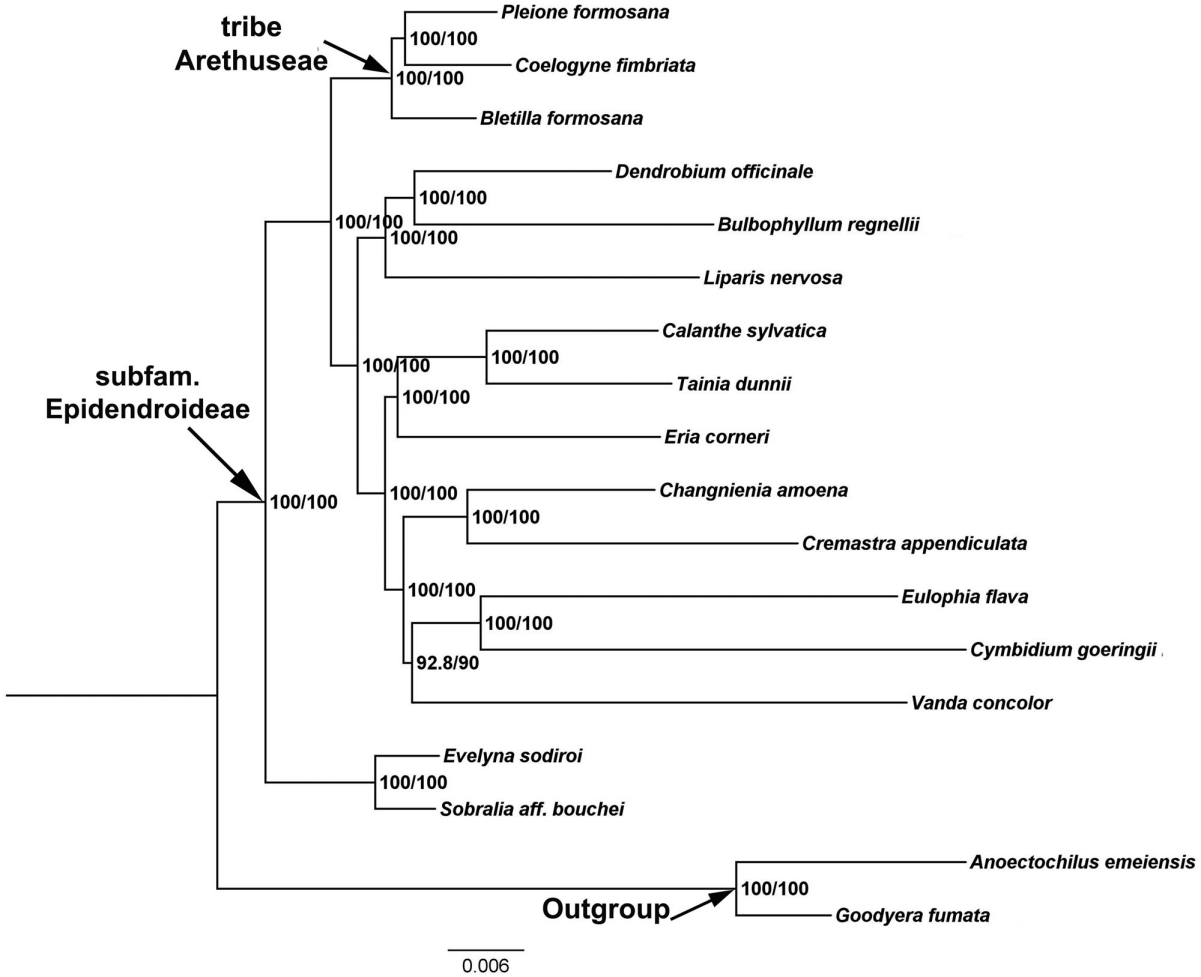
The maximum-likelihood (ML) phylogenetic tree based on the complete chloroplast genome sequences. Numbers at the right of nodes are PP/BPMP. Chloroplast genome accession number used in this phylogeny analysis: *Pleione formosana*:NC_042197; *Coelogyne fimbriata*: MT705722 (the sample in this study);*Bletilla formosana*: NC_045842;*Dendrobium officinale*: NC_024019; *Bulbophyllum regnellii*: NC_048483; *Liparis nervosa*: NC_045896; *Calanthe sylvatica*: NC_044633; *Tainia dunnii*: MN641754; *Eria corneri*: MN477202; *Changnienia amoena*: MN047293; *Cremastra appendiculata*: MH356724; *Eulophia flava*: MK855051; *Cymbidium goeringii*: KT722982; *Vanda concolor*: MK836105; *Evelyna sodiroi*: NC_027266; *Sobralia aff. bouchei*: NC_028209; *Anoectochilus emeiensis*: NC_033895; *Goodyera fumata*: NC_026773.

## Data Availability

The data that support the findings of this study are openly available in GenBank of NCBI at https://www.ncbi.nlm.nih.gov/, reference number MT705722.

## References

[CIT0001] Channell R, Lomolino MV. 2000. Dynamic biogeography and conservation of endangered species. Nature. 403(6765):84–86. 10638757 10.1038/47487

[CIT0002] Clayton D. 2002. The genus Coelogyne: a synopsis. London (UK): Royal Botanic Gardens, Kew.

[CIT0003] Gravendeel B, Chase MW, de Vogel EF, Roos MC, Mes TH, Bachmann K. 2001. Molecular phylogeny of Coelogyne (Epidendroideae; Orchidaceae) based on plastid RFLPS, matK, and nuclear ribosomal ITS sequences: evidence for polyphyly. Am J Bot. 88(10):1915–1927. 21669624

[CIT0004] Jin JJ, Yu WB, Yang JB, Song Y, Yi TS, Li DZ. 2018. GetOrganelle: a simple and fast pipeline for de novo assembly of a complete circular chloroplast genome using genome skimming data. bioRxiv. 256479.

[CIT0005] Katoh K, Standley DM. 2013. MAFFT multiple sequence alignment software version 7: improvements in performance and usability. Mol Biol Evol. 30(4):772–780. 23329690 10.1093/molbev/mst010 PMC3603318

[CIT0006] Kearse M, Moir R, Wilson A, Stones-Havas S, Cheung M, Sturrock S, Buxton S, Cooper A, Markowitz S, Duran C, et al. 2012. Geneious Basic: an integrated and extendable desktop software platform for the organization and analysis of sequence data. Bioinformatics. 28(12):1647–1649. 22543367 10.1093/bioinformatics/bts199 PMC3371832

[CIT0007] Nguyen LT, Schmidt HA, Haeseler AV, Minh BQ. 2015. IQ-TREE: a fast and effective stochastic algorithm for estimating maximum-likelihood phylogenies. Mol Biol Evol. 32(1):268–274. 25371430 10.1093/molbev/msu300 PMC4271533

[CIT0008] Qu X-J, Moore MJ, Li D-Z, Yi T-S. 2019. PGA: a software package for rapid, accurate, and flexible batch annotation of plastomes. Plant Methods. 15(1):50. 31139240 10.1186/s13007-019-0435-7 PMC6528300

[CIT0009] Swarts ND, Dixon KW. 2009. Terrestrial orchid conservation in the age of extinction. Ann Bot. 104(3):543–556. 19218582 10.1093/aob/mcp025 PMC2720663

